# Limited Effect of Dehydrating via Active vs. Passive Heat Stress on Plasma Volume or Osmolality, Relative to the Effect of These Stressors per Se

**DOI:** 10.3390/nu15040904

**Published:** 2023-02-10

**Authors:** Alexandria Davies, Ashley Paul Akerman, Nancy Jane Rehrer, Simon N. Thornton, James David Cotter

**Affiliations:** 1 School of Physical Education, Sport and Exercise Sciences, University of Otago, Dunedin 9016, New Zealand; 2 Faculty of Medicine, Université de Lorraine, Inserm, DCAC, F-54000 Nancy, France

**Keywords:** hypohydration, heat stress, plasma volume, exercise, thirst

## Abstract

The physiological, perceptual, and functional effects of dehydration may depend on how it is incurred (e.g., intense exercise releases endogenous water via glycogenolysis) but this basic notion has rarely been examined. We investigated the effects of active (exercise) heat- vs. passive heat-induced dehydration, and the kinetics of ad libitum rehydration following each method. Twelve fit participants (five females and seven males) completed four trials in randomised order: DEHydration to −3% change in body mass (∆BM) under passive or active heat stress, and EUHydration to prevent ∆BM under passive or active heat stress. In all trials, participants then sat in a temperate-controlled environment, ate a standard snack and had free access to water and sports drink during their two-hour recovery. During mild dehydration (≤2% ∆BM), active and passive heating caused comparable increases in plasma osmolality (P_osm_: ~4 mOsmol/kg, interaction: *p* = 0.138) and reductions in plasma volume (PV: ~10%, interaction: *p* = 0.718), but heat stress per se was the main driver of hypovolaemia. Thirst in DEHydration was comparably stimulated by active than passive heat stress (*p* < 0.161) and shared the same relation to P_osm_ (r ≥ 0.744) and ∆BM (r ≥ 0.882). Following heat exposures, at 3% gross ∆BM, PV reduction was approximately twice as large from passive versus active heating (*p* = 0.003), whereas P_osm_ perturbations were approximately twice as large from EUHydration versus DEHydration (*p* < 0.001). Rehydrating ad libitum resulted in a similar net fluid balance between passive versus active heat stress and restored PV despite the incomplete replacement of ∆BM. In conclusion, dehydrating by 2% ∆BM via passive heat stress generally did not cause larger changes to PV or P_osm_ than via active heat stress. The heat stressors themselves caused a greater reduction in PV than dehydration did, whereas ingesting water to maintain euhydration produced large reductions in P_osm_ in recovery and therefore appears to be of more physiological significance.

## 1. Introduction

Water is the nutrient that is both most abundant and most rapidly turned over in humans. The regulation of body water is dynamic and complex, whereby its volume, composition, and distribution between fluid compartments are all tightly regulated by an array of homeostatic mechanisms—especially osmotic and hydrostatic pressures [1]. A lower-than-normal total body water volume (TBW), commonly referred to as dehydration [2], may arise frequently in recreational exercise and sport. The extent of such dehydration is typically quantified as the change in body mass (∆BM) because this is assumed to accurately represent the ∆TBW both physically and functionally—albeit not without controversy [3,4,5]. Some scientists and international recommendations advise that losses of ≥2% BM loss have deleterious physiological and performance effects and so should be avoided [6], regardless of the primary fluid-regulatory mechanisms, e.g., thirst [7]. However, physiological changes that occur with bodily fluid loss depend not only on the extent of dehydration but also on the process and compartments from which fluid is lost and, therefore, may differ depending on whether dehydration is induced before or during the exercise of interest and, if induced beforehand, how it is induced [8,9,10,11]. For example, dehydration from iso-osmotic fluid loss such as via diuretics preferentially depletes extracellular volume, including blood volume, with less impact on plasma osmolality (P_osm_) and thus provides only limited stimulus for homeostatic correction [10]. Whereas, dehydration from hypo-osmotic fluid loss, such as via heat-induced sweat, causes intracellular dehydration by way of a partial osmosis-driven recovery of the extracellular fluid volume and thus more rapid homeostatic correction via osmotically-stimulated thirst and neuroendocrine responses [2,10]. 

Even for heat-induced dehydration, a long-standing contentious issue is whether different forms of heat stress incur similar physiological effects and hence also similar psychophysical, psychomotor, or cognitive effects. Some studies have reported that dehydration induced by strenuous exercise, and during the exercise rather than before it, appears to have less effect on plasma volume (PV) and extracellular fluid volume relative to other methods of dehydration [11,12,13]. A smaller PV loss for dehydration via strenuous exercise than via more passive heat stress (i.e., rest or light/moderate exercise in the heat) may be due to strenuous exercise causing (i) different hydrostatic and oncotic pressures, which promote fluid ingress into the vascular space [13,14,15], (ii) greater sympathetic nervous system activation and release of associated stress- and fluid- regulating hormones [16,17], and (iii) greater metabolic demand and the associated endogenous production of water via substrate oxidation [18] alongside the release of glycogen-bound water [19,20,21,22].

The effects of methods of dehydration on P_osm_ are potentially of greater importance than those on PV, and both PV and P_osm_ are principal drivers of myriad cardiovascular, metabolic, neuroendocrine, oxidative stress, and performance-related effects of dehydration [1,23,24,25]. The P_osm_ is regulated directly and more tightly than the PV is, strongly eliciting thirst and thus water ingestion, as well as water retention, especially via the release of vasopressin. The P_osm_ also buffers against changes in PV and is a critical determinant of fluid dysregulation [26,27] and thermoregulatory control [28]. The effects of dehydration on both PV and P_osm_ may be attenuated in exercise versus passive heat stress due to the production or release of water via glycogenolysis, particularly during intense exercise, but this remains unresolved; some studies indicate that exercise-induced dehydration buffers against changes in PV or P_osm_ (see above), and hence invalidates the assumption that ∆BM functionally represents ∆TBW, while others find no such evidence [21,22].

Given that the dehydration studies underpinning the ∆BM-based guidelines for exercise and recovery have used heat stress methodologies that often differ markedly from the way athletes dehydrate in training and competition, it is important that research using ecologically valid designs be undertaken to reveal the effects of dehydration both while exercising and as a consequence of exercise [8,18]. This comprised the first two research questions in this study, i.e., whether physiological and psychophysical strain during (question 1) and after (question 2) those forms of heat stress are greater when realistic dehydration is induced via environmental- rather than exercise-heat stress. The study was powered for effects on PV (see Section 2.6), and we hypothesised that passive heat stress would incur more strain on PV and P_osm_, based on previous findings and the potential endogenous water availability of intense exercise. Effects of each method of dehydration during the respective heat stress were compared at 2% loss of body mass, because behaviour would normally stimulate thirst and drinking to prevent further dehydration in free living people when not exercising [29], and a 2% ∆BM is also applicable to a range of exercise settings [30]. Furthermore, the physiological effects of the two methods of dehydration were tested at 3% ∆BM because these are typical methodological contexts to assess the validity of existing literature [2]; this was undertaken in a temperate environment (~26 °C) to allow direct comparisons.

A relevant consideration of the different forms of heat stress is their effects irrespective of dehydration (e.g., on PV, P_osm_, and thirst) and relative to the dehydration effects. Both passive and active heat stress reduce PV and increase P_osm_ [24], but may exert different influences on thirst due to the desiccating effect of exercise hyperpnea. Hence question three was: what are the effects of these stressors per se? This was addressed by examining physiological and psychophysical strain in dehydration and in BM-neutral conditions under the same stressor. Both stressors were hypothesised to cause reduced PV and increased P_osm_ independently of dehydration.

The information above is relevant to the theory and practice of rehydration. Specifically, if glycogenolysis releases a functional quantity of water but the resynthesis of glycogen also takes approximately one day, then it may be imprudent to rehydrate with the recommended 120–150% of ∆BM in the immediate hours of recovery. Furthermore, appetite for sodium and carbohydrate may differ between modes of heat stress. Therefore, question four was whether rehydration behaviour or efficacy (fluid balance) differed between modes of heat stress. We hypothesised that fluid intake and retention would be greater after passive dehydration (due to higher P_osm_, more loss of PV, and no glycogen deficit).

Finally, potential carryover effects of dehydration or other methods of heat stress on fluid regulatory measures 24 h afterward were assessed as a fifth, but secondary, question because the literature is equivocal on this issue (reviewed in [8,31]) and the present study provided the opportunity to address it.

## 2. Materials and Methods

### 2.1. Ethical Approval

The study was conducted in accordance with the Declaration of Helsinki, except for registration in a clinical trials database, and was approved by the University of Otago Human Health Ethics Committee (protocol code H15/095, approved on 21 December 2015).

### 2.2. Participants

Twelve moderately trained ($\dot{V}$O_2_peak > 40 mL/min/kg, physically active for 1 h ≥ 3 d/wk), healthy young adults (five females) volunteered (Table 1) and provided informed a written consent to participate in the current study. No participants were taking medication (excluding the contraceptive pill) during the study.

### 2.3. Experimental Design 

**Figure 1:**
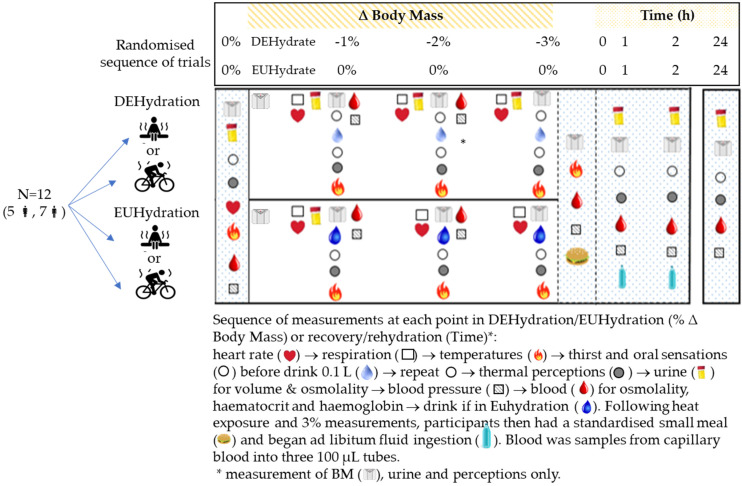
Schematic of experimental design and measures for passive and active dehydration (DEHydration) and euhydration (EUHydration) trials.

The study employed a within-participant, crossover design whereby each participant completed four trials (Figure 1). The order of trials was counterbalanced and randomised using the Latin Squares method, albeit incomplete due to 12 participants and 16 trials. Trials entailed either dehydrating to a mild extent (−3% change in body mass; ∆BM) or drinking to prevent any ∆BM, each within either passive heat stress (~40 °C, 60% RH) or exercise heat stress (cycling intervals at ~90% HR_max_ in 29.5 °C, 50% RH). To conclude each trial, participants drank ad libitum water and/or sports drink for 2 h under supervision, then continued to eat and drink ad libitum for the following 24 h, with monitoring of fluid loss before follow-up measurements at 24 h. 

Trials were separated by at least 1 week for males and by 1–2 or 4 weeks for females (days 16–24, during the self-reported, estimated luteal phase) to minimise the effects of the menstrual phase on fluid regulation [32]. Each participant completed trials at the same time of day to standardise core temperature [33]. Participants were instructed to drink 8 mL/kg and 5 mL/kg of water with dinner and after waking, respectively, to standardise their hydration status prior to each trial. Participants fasted for 12 h before each trial and recorded their food and fluid intake 24 h prior to each trial. Participants were asked to refrain from drinking caffeine or alcohol within 12 h of each trial. They were further requested to undertake no more than 1–2, 1 and 0.5 h of physical activity for 3, 2 and 1 day/s, respectively, prior to each trial, and limit physical activity until after the 24-h follow up measures. Measurements were collected at the times and hydration levels shown in Figure 1, and as described in Section 2.6. Participants were informed about the aim of the study but were blinded to the hypotheses being tested.

### 2.4. Pre-Testing

At least 7 days before the first trial, participants completed an incremental cycling test (Lode B.V., Groningen, The Netherlands) to volitional exhaustion in conjunction with open-circuit spirometry (Cosmed CardioPulmonary Exercise Testing, CosmedSrl, Rome, Italy) to determine peak aerobic power ($\dot{V}$O_2peak_) and maximum heart rate (HR_max_). Baseline measures of BM, urine specific gravity (U_SG_) of the 2nd-morning void, urine colour and thirst were obtained on at least 2 mornings, ≥3 days before or between trials. Height was measured at one of these sessions using a stadiometer.

### 2.5. Experimental Protocol

Upon arrival at the laboratory for each trial, participants voided their bladders before a baseline BM was recorded (±0.02 kg, Wedderburn Scales Ltd., Dunedin, New Zealand), either nude or wearing underwear. The ∆BM from the baseline was used to estimate the level of dehydration after correcting for fluid intake. Following the insertion of a rectal thermistor, a baseline blood sample was collected after being seated for at least 10 min in an upright posture [34]. This posture was standardised for all blood samples, and all blood was arterialised capillary sampling (see Section 2.5). Once all baseline measures were complete (Figure 1), participants entered the environmental chamber and were reweighed on electronic scales to adjust for additional equipment (±0.09 kg, A&D, San Jose, CA, USA).

Passive heat stress entailed sitting sedentary in 40 °C and 60% RH for the first 20–30 min in a hot water bath (~43 °C) to expedite the rise in T_re_ and, in turn, sweating, to achieve a 3% BM loss. Exercising under heat stress entailed high-intensity interval cycling, with a duty cycle of 4 min exercising and 4 min resting, on an electromagnetically-braked cycle ergometer (Velotron Dynafit Pro, RacerMate^®^ Inc., Seattle, WA, USA). Exercise intensity was mostly self-controlled to attain ~90% HR_max_ in the 4th minute. Convection cooling (3.9 m/s) was provided only during intervals, via a large standing fan (655 mm blade, Imasu, Japan), to achieve a 3% BM loss. Workload was replicated within participants for the last interval of each % ΔBM and across both active trials (235 ± 55 W). The workload in the intervening intervals was not strictly monitored or recorded. No set time interval was used for checking ΔBM. 

At each % ∆BM during DEHydration trials (Figure 1), participants ingested a fluid equivalent to ~0.1% ΔBM to uncouple oral sensations from thirst. During EUHydration trials, participants were able to drink tap water (~20 °C) ad libitum from 650 mL bottles (green, opaque) and, at each % ∆BM, were required to ingest water (~20 °C) to replace remaining ΔBM as fast as possible. 

Once a −3% gross ΔBM was achieved, participants returned to the adjacent laboratory (25–27 °C, 33–40% RH) for recording of their corrected ΔBM relative to baseline, i.e., once toweled dry and skin thermistors removed. A capillary blood sample was collected after being seated for ~15–20 min to ensure their posture and physiological state at −3% ∆BM were comparable with baseline conditions and between experimental trials. Participants were provided with a standard meal (250 g) of lasagna (protein: 37 g, fat: 40, CHO: 98 g, sodium: ~500 mg) or macaroni and cheese (protein: 37 g, fat: 40, CHO: 105 g; sodium: ~500 mg) before having ad libitum access to 2500 mL of fluid (~5 °C; water or sports drink, in different bottles: sports drink sodium 540 mg/L, osmolality: ~285 mOsmol/kg), which were weighed before and after to determine the volume of fluid ingested (±0.001 kg, Type 1574, Sartorius, Gottingen, Germany) over the 2-h rehydration period. Participants were then given a post-trial food and urine diary to record food and fluid intake along with urine output (volume, time, and colour). Participants returned 24 h after the start time of their trial for follow up measurements (Figure 1). All nutrition diaries and activity diaries were checked for compliance prior to testing.

### 2.6. Measurements

Rectal temperature (T_re_) was measured using a thermistor (Mallinckrodt Medical Inc., St. Louis, MO, USA), which participants inserted to a depth of ~10 cm. Area-weighted mean skin temperature (T_skin_) was calculated from four right-side sites; chest, the centre of the bicep, the centre of the thigh, and the widest part of the calf [35]. Temperatures were logged at 30-s intervals (Grant 1200 series Squirrel Data Logger, Grant Instruments, Cambridge, UK). Respiratory gases were sampled at 1, 2, 3% ∆BM for 4 min during passive trials and for 8 min (exercise and recovery) during active trials (S-3A Oxygen Analyser and CD-3A Carbon Dioxide Analyser; AEI Technologies, Inc., Bastrop, TX, USA). The RER values > 1.00 or <0.70 were excluded from metabolic analyses. Urine was collected at baseline, 1, 2, 3% ∆BM and 1, 2, and 24 h of recovery, from which urine colour was determined using a urine colour chart (1–8; printed from the internet), and U_SG_ was determined using a hand-held refractometer (Uricon-N, Urine Specific Gravity Refractometer, Atago Co., Tokyo, Japan). Heart rate (HR) was recorded from baseline to 3% ΔBM, and at 1 and 2 h recovery using telemetry from a chest band receiver and stored at 15-s intervals (Polar S810i Heart Rate monitor, Polar Electro Inc., Port Washington, NY, USA). The blood pressure was measured manually and in triplicate with a sphygmomanometer. Thirst was measured on a validated 9-point Likert thirst scale (1: “not thirsty”—9: “very thirsty”) [36], and oral sensations were assessed using the thirst sensation scale (TSS), which contains 6 graded oral sensations associated with thirst [37]. Capillary blood samples were collected into three heparinised 100 μL tubes at baseline, 1, 2, 3% ∆BM and 1, 2 h. Haemoglobin concentration [Hb] (Model OSM3, Radiometer, Copenhagen, Denmark) and hematocrit (Hct) were measured in triplicate. Haematocrit was determined using a custom-made vernier calliper (University of Otago, Dunedin, New Zealand) after being spun for 10 min at 1520 G (Thermo IEC MicroCL 17, radius 8.5 cm). The remaining plasma was stored at −80 °C for later analysis of P_osm_ using a 3-point calibrated (100, 290, and 1000 mOsmol/kg) vapour pressure osmometer (5520 Wescor Vapro, Austin, TX, USA). 

### 2.7. Calculations 

Sample size was calculated based on a pilot study with N = 8 that compared the physiological effects of these two methods of dehydration (Ethical approval number: H14/149). That study found PV to decrease by an average of 5.1% with exercise-induced dehydration, versus 11.8% with passive heat-induced dehydration. The SDwithin was 7.1%, so N = 11 was calculated to provide 80% power for detecting a different PV response at α = 5%. This was larger than the sample indicated by the power analysis of the only similar previous study we were aware of [11]. 

Total energy usage, carbohydrate oxidation, and fat oxidation were estimated using equations derived from Weir [38]. Metabolic water production was quantified from total carbohydrate oxidation and fat oxidation, given that each gram of carbohydrate and fat produces 0.6 and 1.13 g of water, respectively [18]. Glycogen-bound water was accepted to be associated with 3.5 g of water, as estimated across studies [18,19,20]. Respiratory water loss was quantified using equations used in [39] adapted from [40]. Gross ∆BM was determined from the net ∆BM corrected for fluid and food intake [41]. Sweat loss and effective body water loss were determined via equations derived from [41], and relative ∆PV (%) was determined from Hct and [Hb] [42,43]. Twenty-four-hour post-trial dietary intakes were determined using the NZ dietary database (Plant & Food Research Ltd., Auckland, New Zealand). Average daily intakes of macro (fat, carbohydrate, protein, and water) and micro (sodium, potassium) nutrients were determined using the Kai-calculator (Department of Human Nutrition, University of Otago, Dunedin, New Zealand).

### 2.8. Statistical Analysis

For each dependent variable, two-way repeated ANOVA measures of baseline and change scores were performed to examine the effects of dehydration (2 levels: passive and active) and the extent of dehydration (2 levels: EUHydration and DEHydration), for each research question. Tukey’s post hoc comparison tests were used to isolate significant (*p* < 0.05) effects. The extent of dehydration was tested at −2% or −3% ∆BM when examining research questions 1 and 2, respectively. Change scores and homogeneity were assessed using Levene’s test of homogeneity, and the normal distribution of residuals was assessed via the Shapiro-Wilks test. Relations between variables were determined using Pearson’s correlation coefficient. Results are reported as mean ± SD. Supplementary Materials provided at the end of this manuscript will be made available in a data repository (Figshare) with a link provided.

## 3. Results

### 3.1. Compliance

All 12 participants completed the study within five months and adhered to nutrition standardisation requirements. One participant had adverse responses in the passive DEHydration trial for unknown reasons and was moved into the adjacent laboratory intermittently to minimise discomfort. These data were included in the final analysis because physiological variables were collected under standardised environmental conditions and were within three SD of the mean and the range of other participants under the same trial conditions.

### 3.2. Independent Measures (Baselines, ∆BM, Duration, Environmental Conditions)

Baseline measures were comparable before each trial for BM (*p* = 0.730), P_osm_ (*p* = 0.538), thirst (*p* = 0.698), U_SG_ (*p* = 0.650), Hct (*p* = 0.341), and [Hb] (*p* = 0.359; see Supplementary Materials Table S1). Total trial duration, as well as 1, 2, and 3% gross ΔBM, were comparable for all trials (all *p* > 0.270; Table 2). Net ∆BM from baseline to 3% ∆BM was similar between DEHydration trials (*p* = 0.308) and between EUHydration trials (*p* = 0.316; Table 2). Dry bulb temperatures and relative humidities were similar between all conditions at baseline (*p* ≥ 0.493) and within each heat stress method (*p* ≥ 0.973). Dry bulb temperature during 2 h post-trial rehydration was 1.3 ± 0.4 °C (*p* = 0.002) warmer and RH was 6 ± 2% (*p* = 0.027) higher following passive than active heat trials but were similar between DEHydration and EUHydration trials (*p* ≥ 0.298; see Supplementary Materials Table S2).

### 3.3. Plasma Volume and Plasma Osmolality

**Figure 2:**
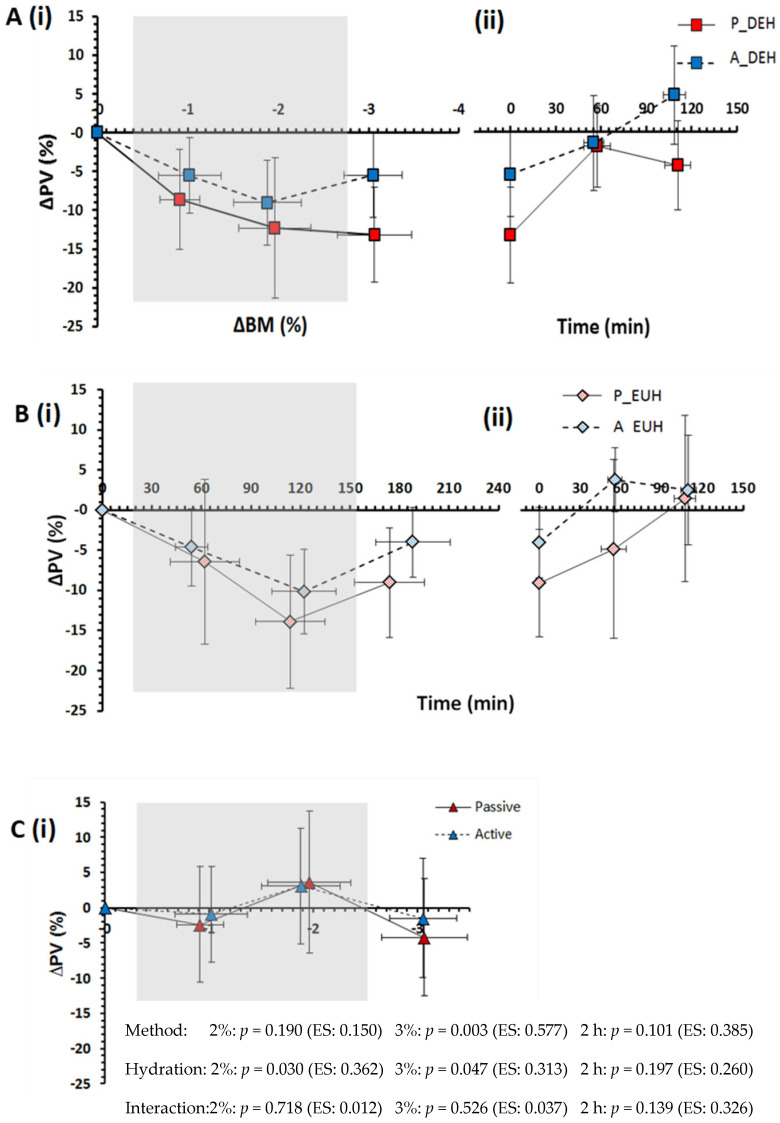
Change in plasma volume (∆PV) during (i) passive DEHydration (Grey area: 40 °C, 60% RH; White area: 25 °C, 35% RH) and active DEHydration (4 min at ~90% HR_max_) in a temperate environment (Grey area: 29.5 °C, 50% RH; White area: 24 °C, 33% RH) to 3% ∆BM with (**A**) no fluid (*p*_DEH; A_DEH); (**B**) fluid intake matched to replace ∆BM (P_EUH;A_EUH) (**C**) controlling for hydration status (passive; active); and (ii) over a 2-h rehydration period following passive and active DEHydration when drinking water and sports drink ad libitum. Data are Mean ± SD for N = 12. ANOVA outcomes for relevant research questions are shown in the bottom panel, including effect size (ES: partial eta, η^2^).

**Figure 3:**
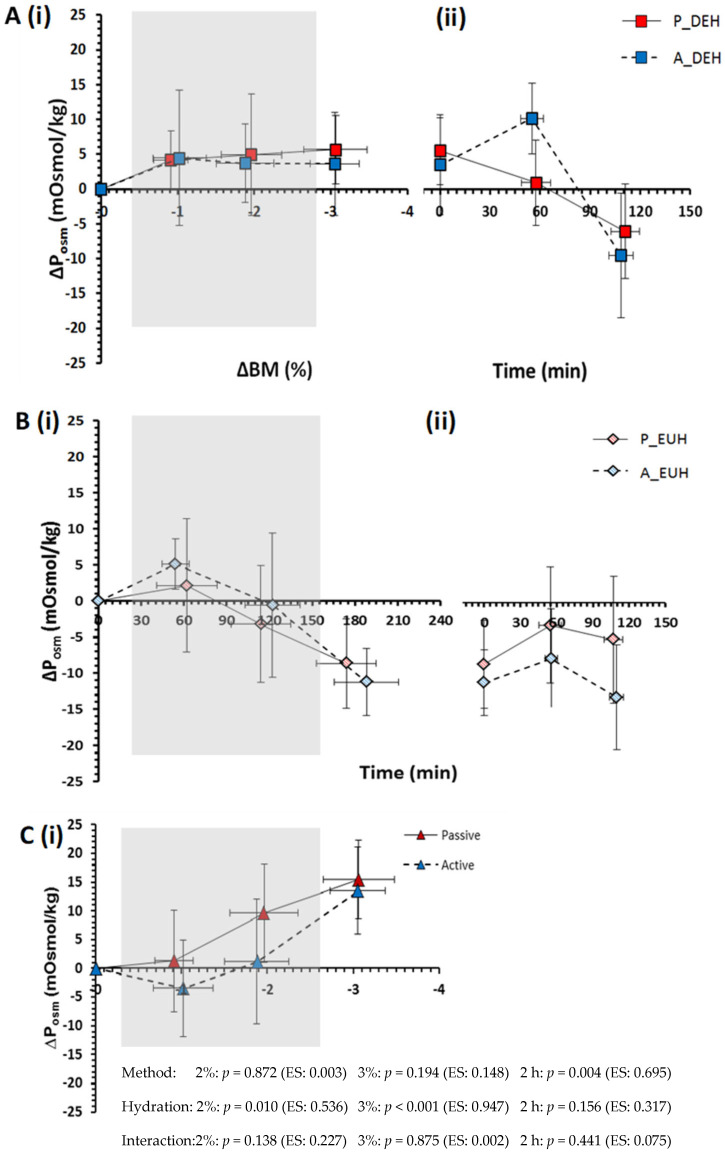
Change in plasma osmolality (∆P_osm_) during (i) passive DEHydration (Grey area: 40 °C, 60% RH; White area: 25 °C, 35% RH) and active DEHydration (4 min bouts at ~90% HR_max_) in a temperate environment (Grey area: 29.5 °C, 50% RH; White area: 24 °C, 33% RH) to 3% ∆BM with (**A**) no fluid (P_DEH;A_DEH); (**B**) fluid intake matched to replace ∆BM (P_EUH;A_EUH) (**C**) controlling for hydration status (passive; active); and (ii) over a 2-h rehydration period following passive and active DEHydration when drinking water and sports drink ad libitum. Data are Mean ± SD for N = 12. ANOVA outcomes for relevant research questions are shown in the bottom panel, including effect size (ES: partial eta, η^2^).

While still exposed to the two forms of heat stress and mild body mass exchanges (i.e., question 1), both passive and active heat stress reduced PV (Figure 2) and increased plasma osmolality (Figure 3), independent of hydration state (Figure 2C and Figure 3C), while DEHydration also caused these effects within both environments. Specifically, DEHydration reduced PV by 3.8 ± 1.6% (Figure 2C) and increased P_osm_ by 5 ± 2 mOsmol/kg (Figure 3C) beyond the effect of the stress itself. 

When subsequently seated in a neutral environment at a 3% nett BM loss (i.e., question 2), PV was reduced to a greater extent in passive DEHydration (Figure 2A), while the rise in P_osm_ was similar between methods (Figure 3A). 

Addressing question 3, the PV was reduced 6.4 ± 1.6% more following passive than active heat stress (*p* = 0.003) and 2.9 ± 1.3% more in DEHydration than EUHydration trials (*p* = 0.047; interaction: *p* = 0.526; Figure 2). The rise in P_osm_ was not significantly higher following passive than active heat stress (*p* = 0.194), but a 15 ± 1 mOsmol/kg difference developed between EUHydration and DEHydration trials (*p* < 0.001, interaction: *p* = 0.875) due to a ~5 mOsmol/kg rise in DEHydration trials and a ~10 mOsmol/kg reduction in EUHydration trials (Figure 3). Thus, P_osm_ was regulated twice as effectively in DEHhydration than EUHydration, regardless of the method of heat stress (averaging ~1% and ~2% ∆P_osm_ per 1%∆ BM for active and passive heat stress, respectively; Figure 3A).

Following the 2-h recovery period (i.e., question 4), recovery of PV showed no reliable difference between active and passive heat stress trials or hydration statuses (Figure 2Aii,Bii), while P_osm_ was 6 ± 1 mOsmol/kg higher following passive than active heat stress trials and showed no reliable difference between DEHydration than EUHydration trials (Figure 3). Finally, at 24 h afterward, PV was not different from baseline, irrespective of the DEHydration method (*p* = 0.331) or occurrence (*p* = 0.139, interaction: *p* = 0.777, Passive heat DEHydration: −2.2 ± 4.8%, Active heat DEHydration: +1.1 ± 5.2%, Passive heat EUHydration: −4.0 ± 10.3%, Active heat EUHydration: −2.0 ± 5.6%). Similarly, P_osm_ at 24 h was not different from baseline (method: *p* = 0.129, occurrence: *p* = 0.153; interaction: *p* = 0.992, for N = 10 due to insufficient sample volume from two participants).

### 3.4. Substrate Oxidation and Mass Exchanges

**Figure 4:**
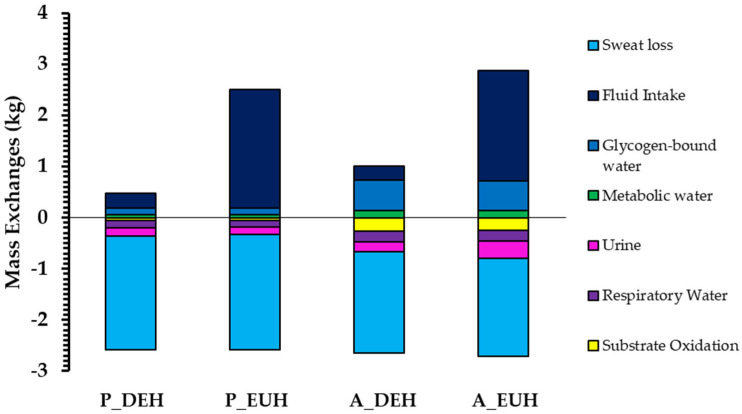
Partitioned mass exchanges during passive heat stress (P_; 40 °C, 60% RH) and active heat stress (A_; 4-min bouts at ~90% HR_max_ in a temperate environment; 29.5 °C, 50% RH), in which fluid intake was negligible (DEH) or matched to prevent any change in body mass (EUH). Note that mass exchanges are in absolute units (kg), not indexed to body mass.

Total energy use (N = 10) was four times higher (*p* < 0.001) and carbohydrate oxidation was five times higher (*p* = 0.001) in active than in passive heat stress trials, but they were similar between DEHydration and EUHydration trials (*p* = 0.928 and 0.906, respectively; interactions: *p* = 0.595 and 0.828, respectively). Estimated volumes of glycogen-water released and metabolic water produced were 457 ± 72 g and 166 ± 14 g, respectively, larger in active than passive heat stress trials (both *p* < 0.001; Figure 4), but were similar between DEHydration and EUHydration trials (*p* = 0.356 and *p* = 0.438, respectively). All sources of remaining mass exchange are shown in Figure 4.

### 3.5. Thirst and Oral Sensations 

**Figure 5:**
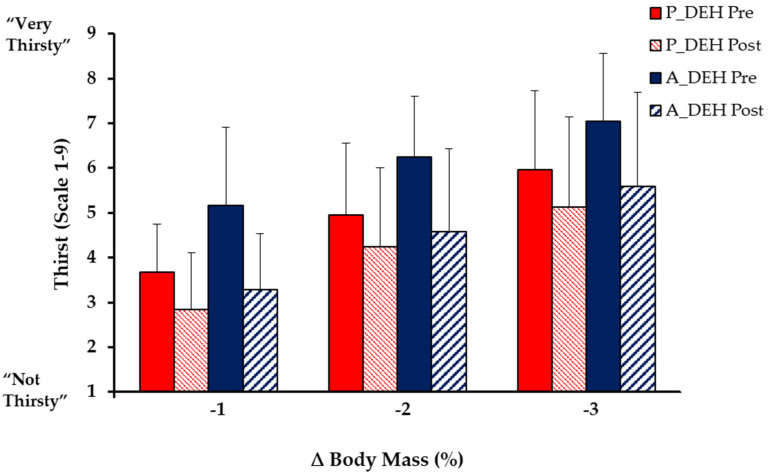
Pre- and post-thirst ratings following ingestion of a standardised small volume of water (0.1% ΔBM) at each of 1, 2, and 3% reductions in body mass during passive DEHydration (*p*_DEH: 40 °C, 60% RH,) and active DEHydration (A_DEH: 4-min bouts at ~90% HR_max_ in a temperate environment; 29.5 °C, 50% RH). Data are mean ± SD for N = 12.

**Figure 6:**
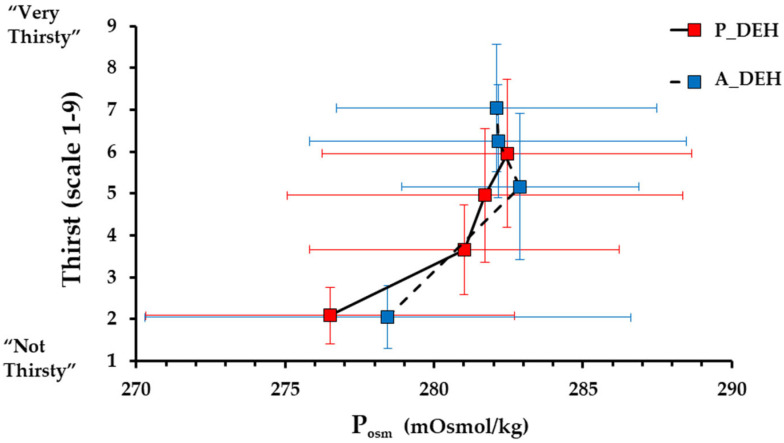
Thirst as a function of plasma osmolality (Posm) during DEHydration from 0 to 3% body mass via passive heat stress (P_DEH; 40 °C, 60% RH) and active heat stress (A_DEH; 4-min bouts at ~90% HR_max_ in a temperate environment; 29.5 °C, 50% RH), in which fluid intake was negligible (0.1 L before each thirst measurement). Data are mean ± SD for N = 12.

From baseline to 3% ∆BM, thirst increased by 3.9 ± 1.9 points for passive DEHydration and by 5.0 ± 1.6 points for active DEHydration (*p* < 0.001) but was not significantly higher for active than passive DEHydration (*p* = 0.161; Figure 5). Mean thirst was strongly correlated with mean ∆BM, mean ∆P_osm_ and mean ∆PV for passive DEHydration (r = 0.980, 0.867 and 0.926, respectively) and for active DEHydration (r = 0.882, 0.744, and 0.712, respectively). The close relation of thirst to P_osm_ was not influenced by the method of dehydration across the range 0 to −3% ∆BM (Figure 6).

From baseline to −3% ∆BM, ‘dry mouth’ increased 2.5 ± 2.3 points during passive DEHydration (*p* = 0.004) and 4.4 ± 2.2 points during active DEHydration (*p* < 0.001), such that at −3% ∆BM, ‘dry mouth’ was 2.3 ± 0.7 points higher in active than passive DEHydration (while still exercising: *p* = 0.026). ‘Dry throat’ was not significantly increased during passive DEHydration (*p* = 0.053), but increased 3.4 ± 2.2 points during active DEHydration, such that at 3% ∆BM, ‘dry throat’ was 2.3 ± 0.9 points higher following active than passive DEHydration (*p* = 0.007). Following ingestion of the token water volume (0.1% ∆BM) at −3% ∆BM, thirst decreased modestly, by 1.3 ± 1.3 points for passive DEHydration (*p* = 0.008; Figure 5) and 1.7 ± 1.4 pts for active DEHydration (*p* < 0.001), as did ‘dry mouth’ (*p* = 0.002 and 0.005), while ‘dry throat’ was reduced by 1.2 ± 1.6 points within active (*p* = 0.027), but not passive DEHydration (*p* = 0.504). 

### 3.6. Heart Rate, Blood Pressure and Thermal Responses

Heart rate in each of the four trials is shown in Supplementary Figures S1 and S2. During the fixed workload exercise in active trials, heart rate was 2 ± 2 b/min higher per % gross ∆BM in DEHydration than in EUHydration (*p* = 0.004), while resting heart rate during passive trials was not significantly affected (*p* = 0.534). 

Baseline systolic and diastolic blood pressures were comparable between conditions (*p* ≥ 0.612). Following heat exposures, when seated in thermoneutrality at −3% ∆BM, neither systolic nor diastolic pressures differed from baseline (*p* ≥ 0.063). At 24 h after exposure, systolic pressure was 4 ± 2 mm Hg lower following DEHydration than EUHydration trials (*p* = 0.035) but did not differ between methods (*p* = 0.947, interaction: *p* = 0.154), whereas an ~3 mm Hg reduction in diastolic pressure showed no carryover effect of DEHydration (*p* = 0.283) or method of heat stress (*p* = 0.819, interaction: *p* = 0.101).

Baseline T_skin_ and T_re_ were not reliably different between conditions (*p* = 0.095 and *p* = 0.657, respectively). The rise in T_re_ from baseline to −3% ∆BM while still within heat stress exposures was not reliably different between passive and active heat stress trials (*p* = 0.055) or between DEHydration and EUHydration trials (*p* = 0.093, interaction: *p* = 0.273, N = 10; Supplementary Figure S3). The T_skin_ rose 2.9 ± 0.7 °C more from baseline to −3% ∆BM in passive than in active heat stress trials (*p* = 0.003) but did not differ significantly between hydration trials (*p* = 0.096, interaction: *p* = 0.731, N = 9). The T_re_ at −3% ∆BM after 20 min thermal recovery in the temperate environment did differ significantly between heat stress method (*p* = 0.113) and hydration status (*p* = 0.441, interaction: *p* = 0.869; Supplementary Figure S4). 

### 3.7. Urine Indices

Urine output decreased (*p* = 0.006) and U_SG_ increased (*p* = 0.016) during DEHydration trials but not EUHydration trials (*p* ≥ 0.189). At −3% ∆BM, urine output and U_SG_ were similar between methods of heat stress (*p* = 0.208 and *p* = 0.220, respectively) and between hydration states (*p* = 0.105, *p* = 0.216, all N = 6 due to micturition difficulty).

### 3.8. Rehydration Fluid Balance

**Figure 7:**
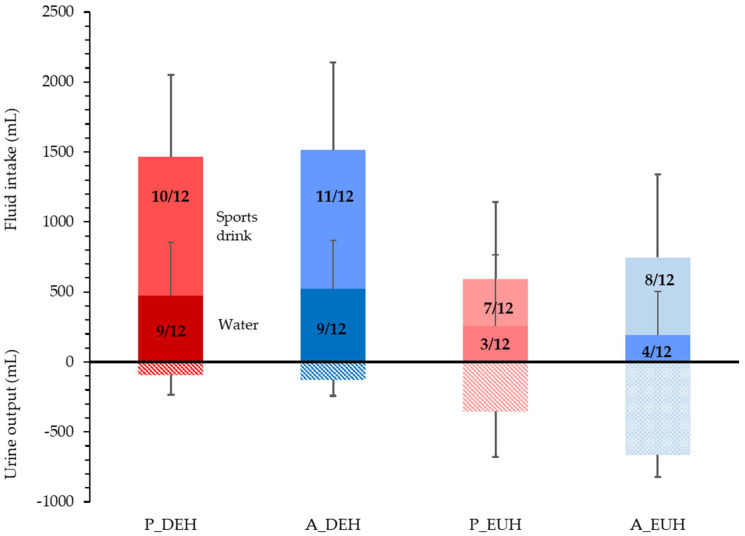
Two-hour fluid balance following passive heat-stress DEHydration (40 °C, 60% RH, P_DEH) and active heat-stress DEHydration (4-min exercise bouts at ~90% HR_max_ in a temperate environment, 29.5 °C, 50% RH, A_DEH). Data are mean for N = 12, with the inset fluid intake bars representing water intake, and the overlaid numbers representing the number of participants ingesting that beverage type.

Fluid intake over the 2-h rehydration period was similar following active versus passive heat stress trials (*p* = 0.272) and was 820 ± 134 mL higher (*p* < 0.001) following DEHydration trials. Approximately three quarters of mass deficit was recovered in 2 h (72 ± 23% and 75 ± 23% following passive and active DEHydration, respectively), and was similar between trials (*p* = 0.306). Ingested volume of sports drink was similar between those trials (*p* = 0.463) and was 547 ± 107 mL higher following DEHydration than EUHydration trials (*p* < 0.001). Total carbohydrate and sodium intake were therefore similar following passive and active DEHydration (*p* = 0.530 and *p* = 0.462, respectively) but carbohydrate was 30 ± 6 g (*p* < 0.001) and sodium 0.3 ± 0.1 g (*p* < 0.001) higher in DEHydration than EUHydration trials (interactions: *p* = 0.433 and 0.500, respectively). Cumulative 2-h urine output differed across DEHydration method and state (interaction: *p* = 0.006), being higher for EUHydration trials (both *p* < 0.001) and for active EUHydration than passive EUHydration (*p* = 0.010). However, 2-h net fluid balance was similarly positive for both passive and active trials (*p* = 0.362; Figure 7) and higher for DEHydration than EUHydration trials (*p* = 0.001, interaction: *p* = 0.449). 

Total carbohydrate intake (including fibre) was similar following passive and active heat stress trials (76 vs. 80 g, *p* = 0.530, Table S3), and was 30 ± 6 g (*p* < 0.001) higher following DEHydration trials than EUHydration trials (interaction effect: *p* = 0.433). Total sodium intake was similar between passive and active heat stress trials (0.9 vs. 0.9 g, *p* = 0.462), but was 0.3 ± 0.1 g (*p* = 0.001) higher for DEHydration than EUHydration trials (interaction effect: *p* = 0.500; Figure S5).

### 3.9. Summary of Key Findings for the Four Main Questions

A summary of the findings is given in Table 3.

### 3.10. Subsequent 24-h Fluid Balances and Nutrient Intakes

The restoration of BM 24-h post-trial did not differ significantly for active versus passive trials (−0.10 vs. −0.24 kg, *p* = 0.242) or for EUHydration versus DEHydration trials (−0.13 vs. −0.20 kg, *p* = 0.585; interaction effects: *p* = 0.088). Twenty-four-hour urine output was 342 ± 148 mL (*p* = 0.043) higher following active trials, and 541 ± 128 mL (*p* = 0.002) higher following EUHydration versus DEHydration trials (interaction effects: *p* = 0.837). Reported water intake (food and fluid) was 621 ± 130 mL (*p* = 0.002) higher following active trials, and 1111 ± 203 mL (*p* = 0.001) higher following DEHydration trials (interaction effects: *p* = 0.072). Salt intake was similar following active trials and passive trials (2.0 vs. 2.4 g, *p* = 0.208), and following DEHydration trials and EUHydration trials (2.6 vs. 1.7 g, *p* = 0.083; interaction effects: *p* = 0.500).

## 4. Discussion

The main purpose of this study was to investigate the effects of passive ambient heat stress versus active exercise heat stress on hydration-related physiological and psychophysical strain(s). The major findings were that (i) passive and active heat stress had comparable effects on PV reduction and P_osm_ elevation with mild dehydration (1–2% ∆BM), as well as thirst, although the sensation of ‘dry mouth’ increased to greater extent during active heat stress (0–3% ∆BM); (ii) passive heat stress caused more reduction in plasma volume when hypohydrated by 3% BM, following the exposures; (iii) preventing dehydration did not change PV kinetics but resulted in substantial reduction in P_osm_ below baseline—a deviation that was also twice as large as the dehydration-induced deviation in P_osm_; (iv) PV and P_osm_ perturbations during both active- and passive- heat-induced DEHydration were determined much less by the dehydration than by the effect of these stressors per se; and (v) ad libitum fluid intake following DEHydration via passive versus active heat stress resulted in a similar net fluid balance and restored PV, despite incomplete replacement of BM. Finally, fluid and cardiovascular regulation one day following these exposures was largely unaffected by the method of heat stress or the occurrence of dehydration, except for a possible, modest reduction in systolic blood pressure following dehydration.

### 4.1. Dehydration

At a 2% BM loss, PV loss was not significantly different under passive versus active heat stress; however, at a 3% BM loss, PV had decreased significantly more from passive than active heat stress-induced DEHydration. In addition, ΔBM accounted for 84% of the variance in PV changes during DEHydration in passive heat stress but only 42% in active heat stress, indicating that the method of dehydration appears to modulate the relationship between PV and BM changes under temperate conditions. Similar findings have been reported by [11,12], who showed that PV decreased ~3–4% more per % ∆BM during passive thermal than during exercise-induced DEHydration. However, it must be highlighted that previous findings are highly varied, with some studies showing no difference between methods [22]. This variation between studies may reflect (i) inconsistencies in blood sampling timing (before vs. during) and (ii) blood sampling posture (supine vs. seated-upright) between studies, which modulate transcapillary forces and consequently, PV and potentially also P_osm_. To minimise these confounding effects in the current study, blood was sampled both during and following each method, with participants in a standardised seated posture. It must be noted, however, that the ∆PV and ∆P_osm_ may be exaggerated at 1 and 2% ∆BM during passive trials due to a partial lower-limb immersion-induced increase in hydrostatic pressure [44], and at −3% ∆BM due to increased capillary filtration in response to a slightly warmer room (by 1.6 °C) and core temperature (T_re_ by 0.4 °C). 

The ∆PV and ∆P_osm_ were similar during active and passive DEHydration, as was thirst, while ratings of dry mouth and dry throat became greater for active DEHydration, and thirst was reduced more after ingesting a token fluid volume (~0.1% ∆BM) in this condition. The desiccating sensations despite similar changes in P_osm_ when under active vs. passive heat stress may reflect the higher ventilation during exercise, which directly dries the mouth, or SNS activation, which indirectly reduces saliva secretion via a decreased blood flow to the salivary glands [45]. It should also be noted that both thirst and oral sensations increased linearly—and prior to 1% ∆BM with a minimal ∆P_osm_—during both passive and active DEHydration. Previous studies have reported similar findings [37,46], with Phillips et al. [29] pointing out that an early rise in oral sensations may provide an anticipatory stimulus to drink, acting in conjunction with renal mechanisms to help maintain fluid balance early in dehydration. Therefore, the current findings further support that (a) non-osmotic or volumetric stimuli, such as SNS activation, may drive fluid regulatory behaviour during exercise and to a greater extent than passive heat stress, and (b) in contrast with common assertions, thirst increases significantly prior to significant changes in P_osm_ or a 3% BM loss. 

Another major finding was that, after controlling for hydration status, passive vs. exercise heat stress had a similar effect on PV and P_osm_. This indicates that the smaller decrease in PV, but comparable increase in P_osm_ following active relative to passive DEHydration, cannot be attributed to a higher total body water, increased free-water availability [17,47,48], or higher hydrostatic or oncotic pressure as a result of exercise [14,15], which is contrary to several studies including the classic study by Pastene et al. [49]. If ‘new water’ cannot account for the maintenance of PV during exercise, the smaller change in PV may be due to a greater redistribution of fluid into the vascular space for exercise relative to passive heat stress. Such a mechanism would support the contention that the body holds a ~2 L fluid reserve [50] that can protect P_osm_ and PV within a body mass loss of ~3% [51]. The demonstration of a larger fluid volume contained within and between tissues, e.g., between collagen layers [52], may account for what has also been considered a third space [53] or reserve for such a flux of water and electrolytes.

A larger fluid shift into the vascular space, in response to active relative to passive DEHydration, could be explained by higher concentrations of vasopressin, angiotensin II, renin and aldosterone, which increase water and sodium reabsorption, and for vasopressin, may induce acute fluid shifts from extravascular compartment into intravascular space [54,55,56]. Although these hormones were not measured in the current study, the role of osmotic and non-osmotic stimuli on vasopressin, renin, and aldosterone release, have been confirmed. Additionally, several studies have reported higher fluid regulatory response during active than during passive DEHydration. For example, Melin et al. [57] reported that PV decreased significantly more during passive than exercise-induced dehydration (~2.5% more per % ∆BM) in conjunction with larger increases in renin, aldosterone and catecholamine concentrations (but not vasopressin). Because P_osm_ provides the primary stimulus for vasopressin secretion [58], this hormone would likely become more concentrated in DEHydration relative to EUHydration trials [59], while the greater SNS activation in response to exercise stress [57,60,61] could induce the non-osmotic release of renin, angiotensin II, aldosterone and perhaps vasopressin. Therefore, a greater fluid shift into the vascular space following (and conceivably during) active, relative to passive DEHydration, might be explained by different sympathetic and hormonal responses to passive and active heat stress, and when in a euhydrated vs. dehydrated state. 

The apparently similar effect of passive vs. exercise-heat stress on PV and P_osm_ might also reflect higher variability introduced in the methodological process of ‘cancelling hydration state’ (i.e., Figure 2C and Figure 3C, respectively). Individual variability will arise from technical error and biological variability in one’s ability to defend PV and P_osm_ during the four trial conditions, which will be modulated by concentrations of fluid regulatory and sex hormones and the rate of fluid absorption into the vascular space [62]. 

When rehydrating to restore BM loss, PV was incompletely restored under passive and exercise-heat stress, yet P_osm_ fell below baseline values and the assumed operating point of osmoregulation (~277 mOsmol/kg; Figure 6). Furthermore, the decrease in P_osm_ was not followed by corrective diuresis, which further supports that rapid ingestion of large quantities of water with reduced renal excretion leads to a significant fall in P_osm_ [63]. These findings agree with previous studies that state fluid-regulatory dysfunction, and the development of exercise-associated hyponatraemia, may be, in part, due to exercise-induced delays in renal filtration [64] and/or inappropriate vasopressin release [17,65]. It is possible that the large decrease in P_osm_ despite the meal consumption may have been due partly to participants’ selection of the sodium-containing beverage for only ~60% of their ingested fluid; however, previous studies indicate that this would have a minimal effect due to the low sodium concentration of sports drinks (18–20 mmol/L). Therefore, the present study supports the notion that aggressive replacement of ∆BM with water during and following exercise seems inappropriate from a homeostatic perspective.

Consistent with previous reports, heart rate increased more in dehydration than in euhydration; however, this heart rate drift was smaller than in previous studies, i.e., heart rate increased ~2 b/min per % ∆BM in this study in contrast to the 4–6 b/min per % ∆BM observed by others [66,67]. The smaller drift in the current study, which occurred despite the concurrent absence of glucose ingestion [68], might be partly attributed to additional convective and evaporative cooling of semi-realistic airflow (3.9 m/s), which may have lessened blood flow and volume in the skin. It must be noted that the intermittent intensity profile of exercise in this study differed from the continuous intensity profile in most previous studies, although this may not explain the current findings because more drift is incurred at higher exercise intensities [67]. 

### 4.2. Rehydration

The extent of ‘voluntary dehydration’ (~75% ∆BM) over the 2-h rehydration period is in agreement with previous studies [69,70,71,72], and the total volume and type of fluid ingested following passive and active DEHydration were almost identical despite differences in ∆PV (Figure 2) and estimated ∆TBW (Figure 4) at −3% ∆BM. The reason behind this similar extent of voluntary dehydration is unclear; however, it presumably reflects a similar rate of decrease in blood variables—especially P_osm_—and oral symptoms, which would reduce thirst and fluid intake (Figure 3 and Figure 5), or could be due to oropharyngeal metering, which is believed to control fluid ingestion prior to fluid absorption to protect from over-hydration [73]. Additionally, the similar 2-h nett positive fluid balance following passive and active trials indicates that fluid retention was similar, and, importantly, that incomplete glycogen resynthesis following active trials did not significantly reduce fluid retention following active DEHydration when drinking ad libitum.

### 4.3. Limitations

Additional limitations to those noted above include the lack of direct knowledge of total body water or any of its component volumes other than plasma volume. These would have required tracer dilution techniques and time, while the volume of glycogen-bound water released during glycogenolysis is more problematic to estimate. Similarly, no hormones were measured to provide more insight into fluid regulatory mechanisms across the four trials. These limitations reflect mostly a limited research budget and demanded priorities placed on participants, along with the need to limit confounding effects between different measures.

### 4.4. Perspectives

One of several contexts for this research relates to international recommendations, such as the American College of Sports Medicine guidelines, which have traditionally asserted that losses of ≥2% BM deleteriously affect physiology and performance, and since thirst cannot be relied upon, fluid replacement strategies should therefore avoid or minimise mass loss during sport and exercise [7,74]. Much of the underpinning research has limitations regarding ecological validity for free-behaving, fit individuals engaged in their usual recreational or competitive physical activity. One validity concern is that dehydration studies typically use heat that is partly-to-wholly passive and is pre-loaded, which thus underestimates some remediating effects of the more complex fluid exchanges occurring during games, field sports, or endurance exercise [8,49]. Nonetheless, the present findings indicate that the water being generated or released during oxidative metabolism may not contribute substantially to PV or P_osm_ allostasis and, thus, may not constitute a serious cause for methodological concern. 

This study also supports previous findings that full rehydration with water (or typical sports drinks) may promote fluid-regulatory dysfunction and thus be inappropriate from a homeostatic perspective [17,51,74,75,76]. For example, when fully replacing ∆BM during exercise, PV loss was incompletely restored, while P_osm_ fell significantly below baseline. Therefore, whilst the validity of BM measurements to gauge hydration state may not depend on the way in which mass is lost, they may similarly not reflect functional dehydration, especially with regard to exercise. Thirst increased linearly with % ∆BM throughout and following both active and passive heat-induced DEHydration, while oral sensory cues were more pronounced during or following active DEHydration. These findings support the merit of behavioural regulation from both osmotic and non-osmotic stimuli for maintaining appropriate hydration in exercise. In addition, following passive- and active-heat-induced DEHydration, ad libitum fluid intakes restored P_osm_ and PV despite the incomplete replacement of ∆BM. Therefore, the present study supports the notion of drinking to thirst and/or ad libitum following exercise.

## 5. Conclusions

The current study demonstrates that passive heat stress (sitting in hot, humid air) and active heat stress (high-intensity interval cycling) had similar impacts on reducing PV and increasing P_osm_. Whereas, dehydration of −2% BM during these forms of heat stress had much less effect on both PV and P_osm_ than the stressors themselves and had stimulated thirst to an extent that may drive behavioural constraint. Following removal from the heat exposures, dehydration-induced PV reduction was larger following passive heat stress, which has implications for the interpretation of hydration studies that used passive or mostly passive heat stress and extrapolate to athletic contexts. Conversely, any contribution of water produced from oxidative metabolism and released from glycogen pools during intense exercise was not statistically evident in the PV or P_osm_ responses within exercise. Nor did these purported intramuscular sources affect the rate of rehydration (e.g., by slowing rehydration early after exercise if glycogen had not been resynthesised). Replacing ∆BM with water during exercise or passive heat stress had little impact on PV during or after exercise, but at 3% BM gross water turnover, it caused P_osm_ to fall twice as far below baseline as P_osm_ had risen during the −3% BM dehydration. Thus, dehydration up to −3% BM may have less impact on PV and P_osm_ than does passive heat stress, active heat stress, or full rehydration with water.

## Figures and Tables

**Table 1:** General Characteristics of Participants.

	N	Age (y)	Height (cm)	Body Mass (kg)	Body Fat ^a^ (%)	$\dot{V}$O_2peak_^b^ (mL/min/kg)
**All**	12	33.5 ± 11.6	172 ± 7	74.4 ± 13.4	20 ± 7	50.7 ± 9.0
**Female**	5	27.2 ± 8.0	167 ± 5	66.2 ± 6.0	24 ± 6	48.4 ± 5.1
**Male**	7	38.0 ± 12.2	176 ± 5	80.3 ± 14.5	17 ± 7	52.6 ± 11.4

Data are mean ± standard deviation. ^a^ determined via bioimpedance; ^b^ determined during cycle ergometry.

**Table 2:** Duration of heat stress required to achieve 3% gross loss of body mass during passive and active heat stress, with negligible fluid replacement (DEHydration), and with water intake matched to fully replace mass loss (EUHydration).

	Trial			
	Passive Heat DEHhydration	Active Heat DEHydration	Passive Heat EUHydration	Active Heat EUHydration
Total duration (min)	179 ± 36	185 ± 12	175 ± 43	187 ± 29
Post BM (kg)	72.6 ± 12.2	72.3 ± 14.3	74.0 ± 15.1	74.6 ± 13.5
Gross ∆BM ^a^ (%)	−3.1 ± 0.6	−3.1 ± 0.3	−3.1 ± 0.5	−3.2 ± 0.5
Net ∆BM ^a^ (%)	−3.1 ± 0.6	−3.1 ± 0.3	−0.1 ± 0.6	−0.0 ± 0.3

∆BM; Change in body mass; ^a^; Mass change measured using scales situated outside environmental chamber; see Figure 1. Data are Mean ± SD for N = 12.

**Table 3:** Summary of findings for the four main research questions.

Research Question	Comparison	Findings			
		ΔP_osm_	ΔPV	Thirst	Uvol
1: Are physiological and psychophysical strains greater when a realistic mild-moderate dehydration is induced via passive, more so than active heat stress, and still in those exposures?	0 vs. −2% BM in respective stressor (e.g., Figure 2A and Figure 3A)	P ≈ A	P ≈ A	P ≈ A	P ≈ A
2: Are fluid-regulatory responses greater when moderately hypohydrated via passive, more so than active heat stress, and now in a non-stressful environment?	0 vs. −3% BM, in a matched setting; resting in a temperate environ. (e.g., Figure 2A and Figure 3A)	P ≈ A	P > A	P ≈ A	P ≈ A
3: What are the effects of these stressors per se? (i.e., by comparing with a BM-neutral condition under the same stressor)	Compare effects across hydration states within each form of heat stress. (e.g., Figure 2B,C and Figure 3B,C)	D < P ≈ A	D < P ≈ A	P < A	D > P ≈ A
4: Does rehydration recovery behaviour or efficacy depend on the method of dehydration?	−3% BM vs. 2 h rehydrate	P ≈ A	P ≈ A	P ≈ A	P ≈ A

P = passive; A = active; D = DEHydration; BM = body mass.

## Data Availability

Data will be made available on Figshare.

## Supplementary Materials

The following supporting information can be downloaded at: https://www.mdpi.com/article/10.3390/nu15040904/s1, Figure S1: Heart rate during Passive heat stress and Active heat stress to 3% ∆BM with negligible fluid intake (Panel A) or ingestion of water matched to replace ∆BM (Panel B); Figure S2. Heart rate during the 4th minute at a constant workrate within exercise intervals in which participants had negligible rehydration or fully replaced mass loss with water ingestion. Figure S3. Change in rectal temperature from baseline during passive DEHydration and active DEHydration to 3% ∆BM with negligible fluid intake (Panel A); or ingestion of water matched to replace ∆BM (Panel B). Figure S4. Change in mean skin temperature from baseline during Passive DEHydration and Active DEHydration to 3% ∆BM with negligible fluid intake (Panel A) or ingestion of water matched to replace ∆BM (Panel B). Figure S5. Total and ad libitum sodium and potassium during a 2-h rehydration period following DEHydration via Passive heat stress or Active heat stress, versus with water intake matched to replace ∆BM. Table S1: Pre-trial values of hydration-related variables before Passive heat stress or Active heat stress in which fluid intake was negligible or matched to prevent any change in body mass. Table S2. Environmental conditions before, during and after passive and active dehydration with and without water intake matched to replace any loss of body mass. Table S3. Intake of macronutrients during 2-h rehydration period following Passive and Active heat stress entailing DEHydration or EUHydration via water intake to replace ∆BM.
