# Supplementary material for: Limited Effect of Dehydrating via Active vs. Passive Heat Stress on Plasma Volume or Osmolality, Relative to the Effect of These Stressors per Se
Source: Nutrients. 2023 Feb 10;15(4):904. doi: 10.3390/nu15040904 (PMC9959915; doi:10.3390/nu15040904)
Supplement: Supplementary file 1 [file nutrients-15-00904-s001.zip › Supplementary Figures_Proof corrected.pdf]

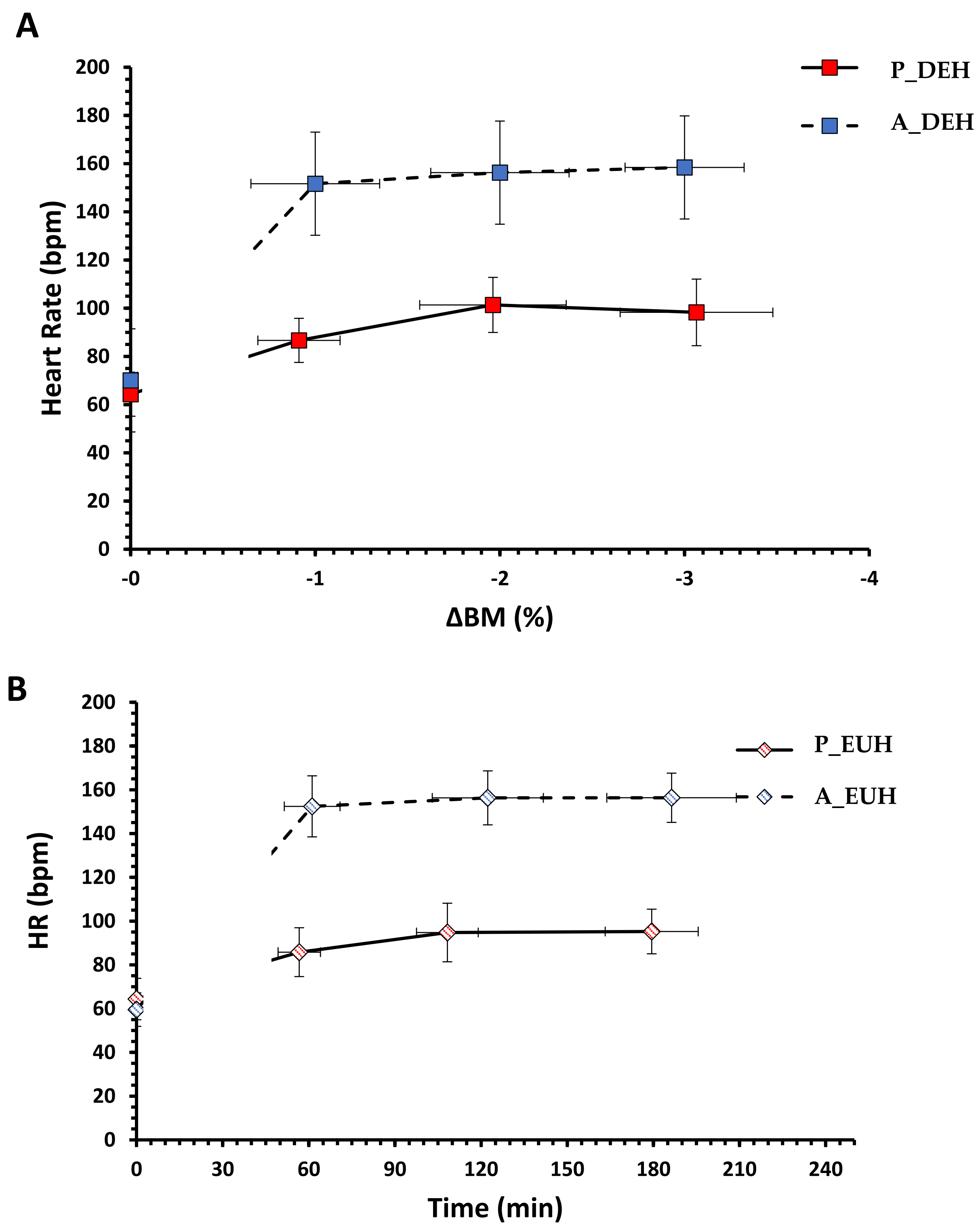

Figure S1. Heart rate (HR) during Passive heat stress (40˚C, 60% RH) and Active heat stress (repeated 4-min exercise intervals at ~90% HR_{max} in a temperate environment (29.5˚C, 50% RH)) to 3% ΔBM with negligible fluid intake (P_DEH; A_DEH; Panel A) or ingestion of water matched to replace ΔBM (P_EUH; A_EUH; Panel B). Data are Mean ± SD for *N* = 12.

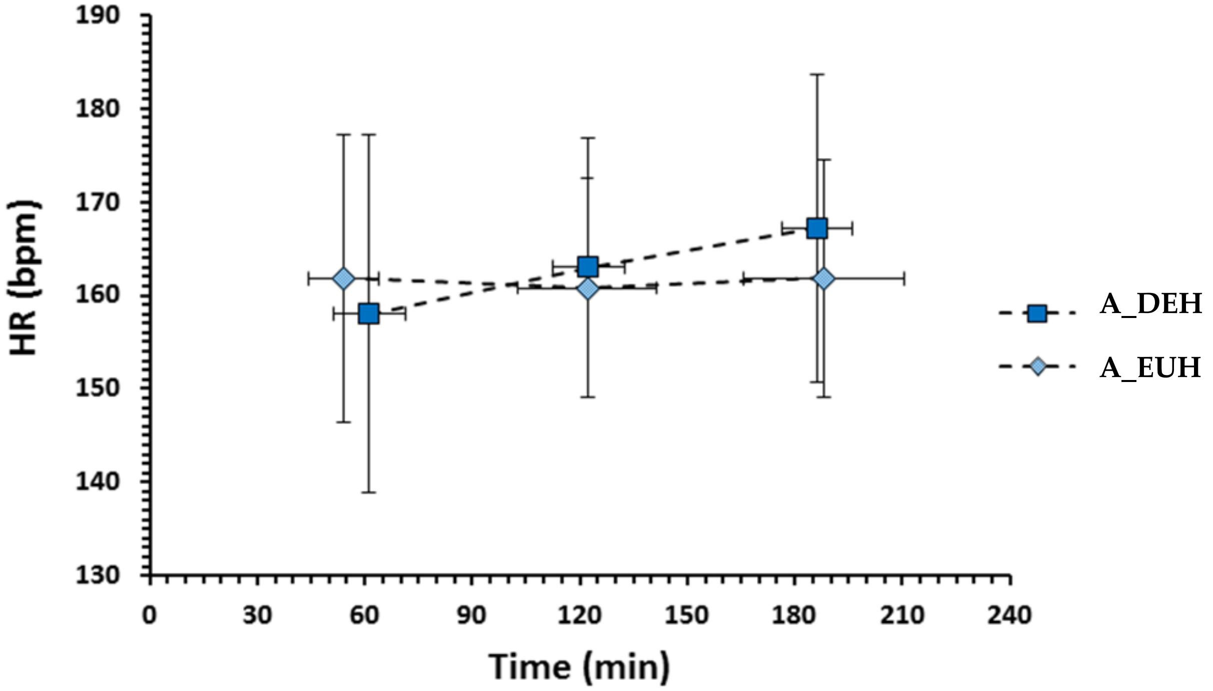

Figure S2. Heart rate (HR) during the 4th minute at a constant workrate within exercise intervals in which participants had negligible rehydration (A_DEH) or fully replaced mass loss with water ingestion (A_EUH). Data points are at each 1, 2 and 3% gross ΔBM. Work rate was fixed and replicated across intervals and across trials for each participant (Mean 235 W for the cohort). Data are Mean ± SD for *N* = 12.

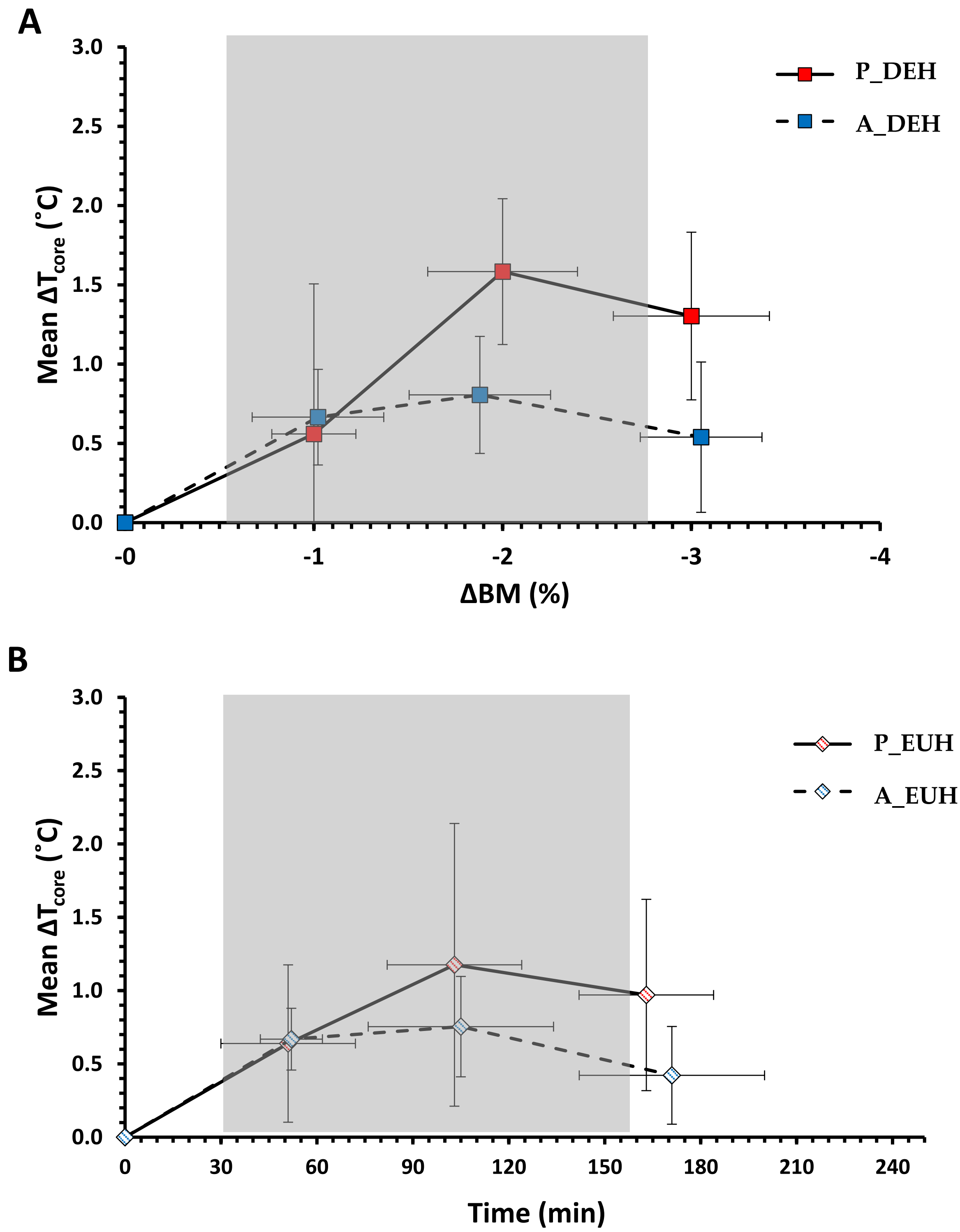

Figure S3. Change in rectal temperature from baseline (ΔT_{core}) during passive DEHydration (P-DEH: 40˚C, 60% RH) and active DEHydration (repeated 4-min exercise intervals at ~90% HR_{max} in a temperate environment (Grey area: 29.5˚C, 50% RH)) to 3% ΔBM with negligible fluid intake (P_DEH; A_DEH; Panel A); or ingestion of water matched to replace ΔBM (P_EUH;A_EUH; Panel B). The grey area represents the respective heat stress periods. The final data point is following 15 min seated in a temperate environment (see Section 3.2). Data are Mean ± SD for *N* = 10.

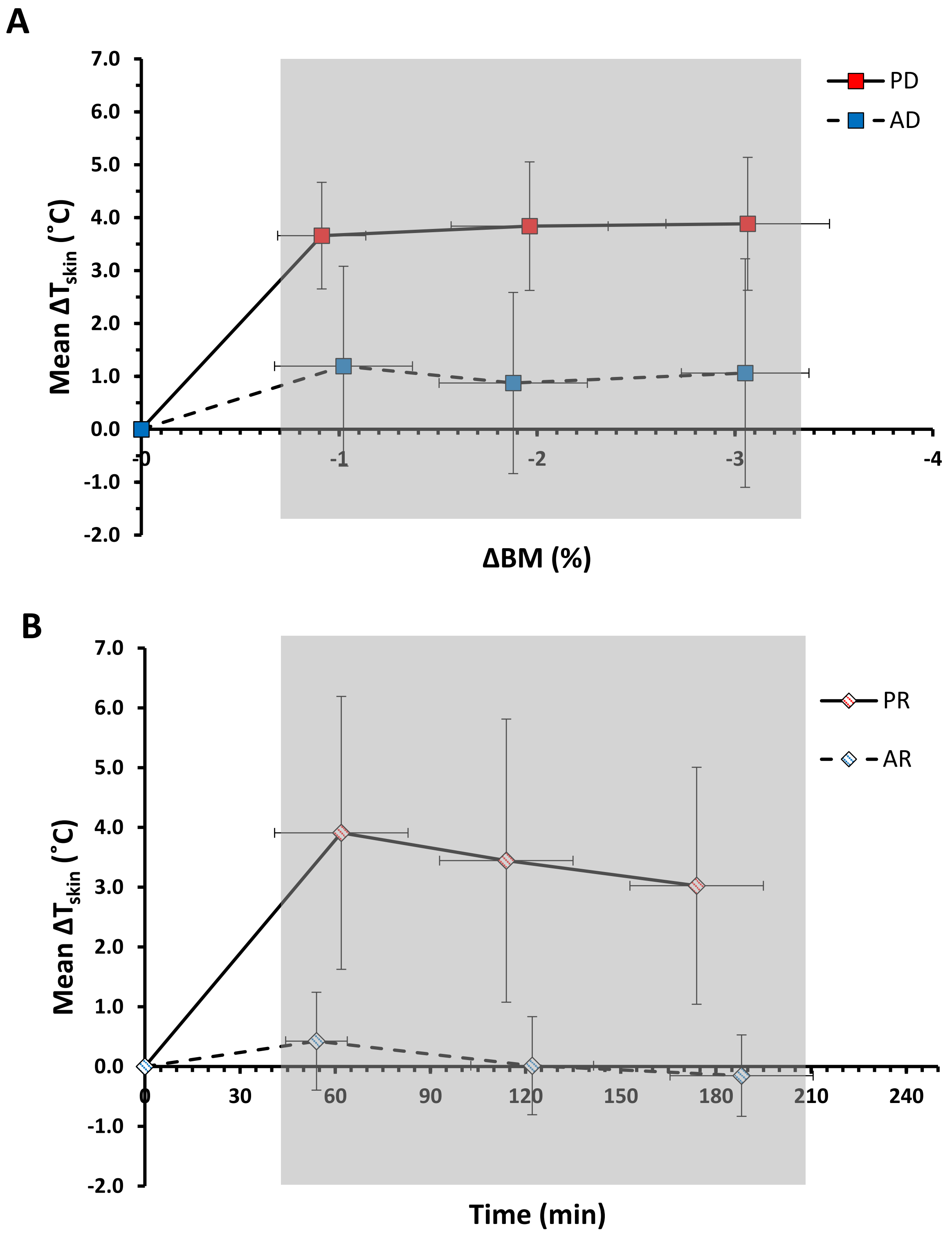

Figure S4. Change in mean skin temperature from baseline (ΔT_{skin}) during Passive DEHydration (P-DEH: 40˚C, 60% RH) and Active DEHydration (repeated 4-min exercise intervals at ~90% HR_{max} in a temperate environment (Grey area: 29.5˚C, 50% RH)) to 3% ΔBM with negligible fluid intake (P_DEH; A_DEH; Panel A); or ingestion of water matched to replace ΔBM (P_EUH;A_EUH; Panel B). The grey area represents the respective heat stress periods (see Section 3.2). The skin thermistors were removed for exiting the climate chamber so no recovery data are obtained. Data are Mean ± SD for *N* = 9.

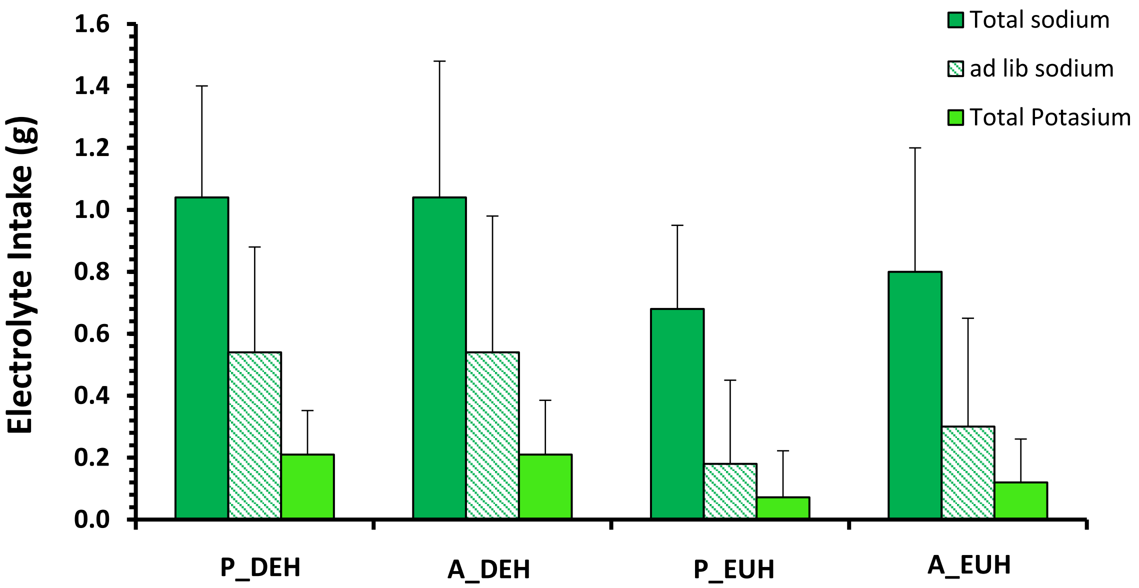

Figure S5. Total and *ad libitum* sodium and potassium during a 2-h rehydration period following DEHydration via Passive heat stress (P_DEH: 40˚C, 60% RH) or Active heat stress (A_DEH: repeated 4-min exercise intervals at ~90% HR_{max} in 29.5˚C, 50% RH), versus with water intake matched to replace ΔBM (P_EUH and A_EUH). Data are Mean ± SD for *N* = 12.
