# Supplementary material for: Limited Effect of Dehydrating via Active vs. Passive Heat Stress on Plasma Volume or Osmolality, Relative to the Effect of These Stressors per Se
Source: Nutrients. 2023 Feb 10;15(4):904. doi: 10.3390/nu15040904 (PMC9959915; doi:10.3390/nu15040904)
Supplement: Supplementary file 1 [file nutrients-15-00904-s001.zip › Supplementary Tables_Proof Corrected.pdf]

Table S1. Pre-trial values of hydration-related variables before Passive heat stress (P_; 40˚C, 60% RH), or Active heat stress (A_; 4-min bouts at ~90% HRmax in a temperate environment; 29.5˚C, 50% RH), in which fluid intake was negligible (DEH) or matched to prevent any change in body mass (EUH).

	**Trial**			
	P_DEH	A_DEH	P_EUH	A_EUH
Pre BM (kg)	74.6 ± 13.3	74.2 ± 14.6	74.1 ± 13.5	74.7 ± 13.6
Hct (%)	44 ± 4	45 ± 4	45 ± 5	45 ± 4
[Hb] (g/L)	15.6 ± 1.4	16.0 ± 1.2	15.8 ± 1.5	15.6 ± 1.2
P_{osm} (mOsmol/kg)	277 ± 6	278 ± 8	280 ± 4	276 ± 5
U_{SG}	1.013 ± 0.009	1.013 ± 0.007	1.019 ± 0.004	1.014 ± 0.004
Thirst (1-9)	2.1 ± 0.7	2.0 ± 0.8	2.5 ± 1.3	2.3 ± 1.3
TS (1-13)	6.9 ± 1.7	7.8 ± 1.1	7.6 ± 1.4	7.1 ± 0.6
TD (1-10)	1.7 ± 1.7	1.2 ± 1.1	1.8 ± 1.5	1.1 ± 0.3

Pre BM: Pre-trial body mass; Hct: Haematocrit; [Hb]: haemoglobin concentration; P_{osm}: plasma osmolality; U_{SG}: urine specific gravity; TS: thermal sensation; TD: thermal discomfort. P_DEH: Passive heat stress DEHydration; A_DEH: Active heat stress DEHydration; P_EUH: Passive heat stress EUHydration; A_EUH: Active heat stress EUHydration. Data are Mean ± SD for N = 12.

Table S2. Environmental conditions before, during and after passive and active dehydration with and without water intake matched to replace any loss of body mass.

	**Trial**			
	P_DEH	A_DEH	P_EUH	A_EUH
Baseline temp. (˚C)	23.4 ± 2.1	24.2 ± 1.5	23.5 ± 1.1	23.0 ± 1.1
RH (%)	34 ± 9	31 ± 8	33 ± 7	32 ± 6
Trial temp. (˚C)	40.0 ± 0.0	29.5 ± 0.1	40.0 ± 0.0	29.5 ± 0.2
RH (%)	60 ± 1	48 ± 7	60 ± 0	50 ± 0
Rehydration temp. (˚C)	26.7 ± 1.7	25.2 ± 1.1	26.9 ± 1.2	25.4 ± 1.2
RH (%)	40 ± 4	34 ± 3	37 ± 4	33 ± 6

RH: relative humidity; P_DEH: Passive heat stress DEHydration; A_DEH: Active heat stress DEHydration; P_EUH: Passive heat stress EUHydration; A_EUH: Active heat stress EUHydration Data are Mean ± SD for N = 12.

Table S3. Intake of macronutrients during 2-h rehydration period following Passive and Active heat stress entailing DEHydration or EUHydration via water intake to replace ΔBM.

	Trial			
	P_DEH	A_DEH	P_EUH	A_EUH
Total Energy Intake (kJ)	2470 ± 652	2462 ± 802	1818 ± 481	2044 ± 635
Total Energy Intake (kcal)	591 ± 156	589 ± 192	435 ± 115	489 ± 152
Total CHO (g)	94 ± 37	92 ± 44	57 ± 28	69 ± 36
Ad libitum CHO (g)	56 ± 37	53 ± 44	19 ± 28	31 ± 36
Total Fat (g)	15.8 ± 0	15.8 ± 0	15.8 ± 0	15.8 ± 0
Total Protein (g)	15.2 ± 0	15.2 ± 0	15.2 ± 0	15.2 ± 0

P_DEH: Passive heat stress; DEHydration; A_DEH: Active heat stress DEHydration; P_EUH: Passive heat stress EUHydration; A_EUH: Active heat stress EUHydration. Data are Mean ± SD for N = 12.
